# High-Value Bioconversion of Ginseng Extracts in Betaine-Based Deep Eutectic Solvents for the Preparation of Deglycosylated Ginsenosides

**DOI:** 10.3390/foods12030496

**Published:** 2023-01-20

**Authors:** Wenhua Yang, Qiuya Gu, Jianli Zhou, Xiaobo Liu, Xiaobin Yu

**Affiliations:** 1 Key Laboratory of Industrial Biotechnology, Ministry of Education, School of Biotechnology, Jiangnan University, Wuxi 214000, China; 2 Guizhou Province Key Laboratory of Fermentation Engineering and Biopharmacy, School of Liquor and Food Engineering, Guizhou University, Guiyang 550003, China; 3 School of Environmental and Biological Engineering, Nanjing University of Science and Technology, Nanjing 210094, China

**Keywords:** deep eutectic solvents, deglycosylated ginsenosides, biocatalysis, aqueous two-phase system, characterization

## Abstract

Deep eutectic solvents (DES), as a green alternative to traditional organic solvents in biocatalysis, not only activate proteins but even increase the efficiency of enzymatic reactions. Here, DES were used in a combinatorial enzyme-catalyzed system containing β-glucosidase BGLAt and β-galactosidase BGALAo to produce deglycosylated ginsenosides (De-g) from ginseng extracts (GE). The results showed that DES prepared with betaine and ethylene glycol (molar ratio, 1:2) could significantly stimulate the activity of the combinatorial enzymes as well as improve the acid resistance and temperature stability. The DES-based combinatorial enzyme-catalyzed system could convert 5 g of GE into 1.24 g of De-g (F_1_, F_2_, 20 (*S*)-PPT, and CK) at 24 h, which was 1.1 times that of the buffer sample. As confirmed by the spectral data, the changes in the conformations of the combinatorial enzymes were more favorable for the binding reaction with the substrates. Moreover, the constructed DES-based aqueous two-phase system enabled the recovery of substantial amounts of DES and De-g from the top phase. These results demonstrated that DES shows great application as a reaction solvent for the scale-up production of De-g and provide insights for the green extraction of natural products.

## 1. Introduction

Ginseng has long been a highly valued herb around the world for maintaining physical vitality and prolonging life. It has been widely reported that the main active components in ginseng are ginsenosides [1]. According to the degree of deglycosylation, ginsenosides can be divided into major ginsenosides (Ma-g) and deglycosylated ginsenosides (De-g). Among them, De-g are obtained by the deglycosylation of Ma-g, which are absent or very low in the natural ginseng plant [2]. De-g (e.g., ginsenosides F_1_, F_2_, CK, 20 (*S*)-PPT, etc.) have better membrane permeability and bioavailability than Ma-g [3]. It was found that De-g have tremendous anti-cancer, anti-inflammatory, and neuro-immune applications [4,5,6,7,8]. With the increasing demand for De-g, it has become a research direction for scholars to produce such metabolites, which are not present in ginseng plants or are present only at very low levels.

Currently, physical and chemical methods are still the main options for the industrial preparation of De-g [9,10]. Substantial amounts of De-g Rd and 20 (*S*)-Rg_3_ can be obtained from ginseng extracts (GE) by combining β-xylosidase Tpexyl3 and β-glucosidase Tpebgl3 [11]. This combinatorial enzymatic catalytic strategy is a clean, efficient, and stable alternative to prepare De-g. In addition, with the development of green chemistry, more attention has been paid to green and safe solvents as a medium for enzymatic reactions [12]. Deep eutectic solvents (DES) are simple compounds with 100% atomic economy as an alternative to conventional organic solvents in enzymatic reactions [13]. Notably, DES not only activate and stabilize proteins (e.g., β-glucosidase) but also increase their reaction efficiency [14,15]. Therefore, the use of green and economical DES as an enzymatic reaction solvent is advantageous for the scale-up production of De-g.

The conventional method for extracting ginsenosides is based on certain concentrations of ethanol combined with heat-reflux, shaking, or ultrasound-assisted extraction [16], which involve a large volume of volatile organic solvent and a long extraction time. Moreover, extraction is often conducted at a high temperature, which requires more energy. Li et al. developed a DES-based aqueous two-phase system (ATPS) for the rapid recovery of eight ginsenosides from an injection [17]. Xu et al. developed a DES-based ATPS that recovered 91.73% of the lysozyme from the system, and the recovered enzyme could also be used for actual sample analysis [18]. Therefore, the combination of DES and ATPS may be a rapid and sensitive method for the enrichment of ginsenosides and biocatalysts.

In our former study, the β-glucosidase BGLAt from *Aspergillus tubingensis* was found to be a good candidate for converting Rb_1_, Rb_2_, Rb_3_, and Rc into Rd and F_2_ into CK. In addition, β-galactosidase BGALAo from *Aspergillus oryzae* could convert Rd into F_2_ (Figure S1). Therefore, using the advantages of both enzymes, this study aimed to establish a DES-based combinatorial enzyme catalytic system for the high-value conversion of GE for the preparation of De-g. Twenty-four DES were screened to assess the effect of DES on the combinatorial enzymes, and the effects of DES on the enzymatic properties of the combinatorial enzymes were explored. Based on the above study, a DES-based combinatorial enzyme-catalyzed system was established to prepare De-g. Then, a DES-based ATPS was constructed for the green extraction of ginsenosides. In addition, the effects of DES on the conformation of the combinatorial enzymes were analyzed using spectral characterization. The aim of this study was to high-value transform GE into De-g and to provide insights into the green extraction of natural products.

## 2. Materials and Methods

### 2.1. Materials

β-glucosidase BGLAt (30 U/mg) purified from *A. tubingensis* extracellular protein and β-galactosidase BGALAo (17 U/mg) purified from *A. oryzae* extracellular protein were stored at −80 °C (Figure S1A). Ginsenosides standards were purchased from Chengdu Must Biotechnology Co., Ltd. (Chengdu, Sichuan, China), with a purity of 98%. Ginseng extracts (GE) extracted from the stems, leaves, and roots of Panax ginseng (the purity of ginsenoside is 80%) were obtained from Xi’an Tianxingjian Natural Bio-products Co., Ltd. (Xi’an, Shanxi, China). Choline chloride (99%), betaine (99%), and other chemicals were obtained from Sigma-Aldrich (Shanghai, China).

### 2.2. Synthesis of DES

DES were prepared using a heating method as previously described [19]. Briefly, hydrogen bond acceptors (HBA) and hydrogen bond donors (HBD) were composed according to the ratio in Table 1. The mixtures were heated at 80 °C until a homogeneous and transparent liquid was formed. The prepared DES were cooled down to room temperature and stored in a vacuum desiccator until further use.

### 2.3. Enzyme Stability in DES

The effects of DES (24 synthetic DES) on the activity and stability of BGLAt and BGALAo were evaluated in a citrate buffer (20 mM, pH 6.0) containing 10 wt % DES, respectively. Then, the effects of DES concentration (10, 20, 30, 40, 50, and 60 wt %) on the glycosidase hydrolysis system were further evaluated by selecting the DES concentration that significantly activated the enzyme. In all tests, as above, the mixture was incubated at room temperature for 30 min, and then the enzyme activity was measured under standard assay conditions (as described in Section 2.4). Catalytic activity without adding DES was used as the control, and the activity was defined as 100%.

Furthermore, the effects of pH and temperature on the enzymatic activity of BGLAt and BGALAo incubated in the buffer containing 10 wt % DES (Bet: EG, 1:2) were investigated. The effect of pH on enzyme activity was assessed using DES/Gly-HCL buffer (pH 2.5–3.5, 20 mM), DES/acetate buffer (pH 4.0–5.5, 20 mM), and DES/citrate buffer (pH 6.0–8.0, 20 mM). Further, the enzymes were pre-incubated in the corresponding DES/buffer without any substrate at 50 °C for 1 h to determine the pH stability. Moreover, the optimal temperatures of BGLAt and BGALAo were determined by measuring their activity at 30–65 °C in the citrate buffer (20 mM, pH 6.0) containing 10 wt % DES (Bet: EG, 1:2). Meanwhile, the thermal stabilities of BGLAt and BGALAo were investigated by incubating the samples at different temperatures (30–65 °C) for 0, 0.5, 1, 2, and 4 h in the citrate buffer (20 mM, pH 6.0) containing 10 wt % DES (Bet: EG, 1:2), respectively. The enzyme activity assay conditions for the above experiments were consistent with the standard assay conditions except for the studied variables.

### 2.4. Enzyme Activity Analysis

The enzymatic activity of BGLAt was measured by referring to the pNPG method [20]. Fifty μL of the enzyme solution was mixed with 100 μL of 5 mM *p*-nitrophenyl-β-D-glucopyranoside, which reacted at 50 °C for 30 min. Then, 1 mL of 1 M Na_2_CO_3_ was added to terminate the reaction. Absorbance data were obtained using a microplate spectrophotometer at 400 nm. One unit of enzymatic activity was defined as the amount of enzyme required to release 1 μmol of *p*-nitrophenol per min under assay conditions. The enzymatic activity of BGALAo was analyzed using a β-galactosidase assay kit (Beyotime, Shanghai, China).

### 2.5. Enzymatic Conversion of Ginsenosides

One mL citrate buffer (20 mM, pH 6.0) containing 10 wt % DES (Bet: EG, 2:1) was used as the reaction medium. In addition, the standard reaction mixture (1 mL) containing 10 g/L BGLAt, 10 g/L BGALAo, and 5 g/L GE, reacted at 50 °C for 24 h. To obtain the optimal conversion conditions for producing De-g, the effects of reaction factors, including the concentration of GE (0.5, 1.0, 2.5, 5.0, and 10.0 g/L), the transformation duration (0, 1, 2, 4, 6, 9, 12, 24, 36, 48, 60, and 72 h), and the dosage of BGLAt (1, 5, 10, 20, 50, and 100 g/L), were investigated sequentially.

### 2.6. Preparation of Phase Diagrams

The phase diagram of DES/K_2_HPO_4_ was determined by the cloud point titration method at room temperature [21]. A certain amount of DES-buffer (citrate buffer (20 mM, pH 6.0) containing 50 wt % DES (Bet: EG, 2:1)) was loaded into a test tube, and 800 g/L K_2_HPO_4_ solution was added drop by drop and shaken until the mixture became cloudy. Then, the deionized water was added to make the solution clarified, and the above procedure was repeated to acquire adequate data. Phase diagrams were constructed with the concentration of DES and salt as indicators.

### 2.7. DES-Based ATPS

At first, 1 mL citrate buffer (20 mM, pH 6.0) containing 10 wt % DES (Bet: EG, 2:1) was added to a tube. The reaction mixture (1 mL), containing 50 g/L BGLAt, 10 g/L BGALAo, and 5 g/L GE, reacted at 50 °C for 24 h. Then, DES (Bet: EG, 2:1; a final concentration of 30 wt %) and K_2_HPO_4_ (a final concentration of 60 wt %) were added to the former solution to construct ATPS. Subsequently, the mixtures were shaken for 6 h at 200 rpm at 25 °C. The mixtures were centrifuged for 5 min at 8000 rpm/min to allow the boundary between the upper and bottom phase to become clear. After extraction, the volume of the top and bottom phases was recorded. Finally, the upper phase was treated and injected into the HPLC for the analysis of ginsenosides. To obtain the optimal recovery concentration of ginsenosides, the effects of DES (Bet: EG, 2:1) concentration (28, 30, 32, 34, 36, 38, and 40 wt %), K_2_HPO_4_ concentration (60, 65, 70, 75, and 80 wt %), and extraction time (0.25, 0.5, 1, 2, 4, 6, 12, and 24 h) were investigated sequentially.

The upper phase containing the DES was separated for recycling the DES. The potential of recycling systems was evaluated by reusing the recovered DES for enzymatic reactions. DES (Bet: EG, 2:1; a final concentration of 30 wt %), K_2_HPO_4_ (a final concentration of 60 wt %), 50 g/L BGLAt, 10 g/L BGALAo, and 5 g/L GE were added at each cycle. The recovery rate of deglycosylated ginsenosides in the ATPS was calculated using the following equation according to Han et al. [22]:

(1) $$\mathrm{Recovry}\;\mathrm{rate}=\left(C_{T\;}\times V_{T}\right)/\left(C_{E}\times V_{E}\right)$$

*V_T_* represents the top phase volume containing ginsenosides, *V_E_* represents the extract volume, and *C_T_* and *C_E_* represent the ginsenosides concentration of the top phase and theoretical extract.

### 2.8. Quantitative Analysis of Ginsenosides

The samples were extracted with an equal volume of n-butanol, and the top phase (water-saturated n-butanol fraction) was evaporated and re-dissolved with 100% methanol. Then, the solutions were filtered through a 0.22 μm filter membrane and analyzed for ginsenosides by an Agilent 1260 HPLC system (Agilent Technology, Palo Alto, CA, USA) equipped with a Sepax GP-C18 column (4.6 mm × 250 mm, 5 μm). The assay procedure is consistent with previous study [23].

### 2.9. Structural Characterization of Enzymes

An F-2700 fluorescence spectrometer (Hitachi, Japan) was used for fluorescence spectrum analysis. Protein samples (0.5 µmol·L^−1^ BGLAt or BGALAo) were incubated for 30 min in citrate buffer (20 mM, pH 6.0) or citrate buffer (20 mM, pH 6.0) containing 10 wt % DES (Bet: EG, 2:1), respectively. After incubating, the enzyme solution was transferred into a 1 × 1 cm quartz cuvette for fluorescence measurements. The samples were excited at 280 nm, and the emission was registered between 300 and 500 nm. Both the excitation and emission slits were set to 5 nm [24]. For each measurement, the respective solvent was used as blank for background subtraction. All fluorescence measurements were carried out at 25 °C.

A Jasco-1700 circular dichroism (CD) spectrometer (Jasco, Tokyo, Japan) was used for CD analysis. Protein samples (BGLAt and BGALAo) were incubated for 30 min in citrate buffer (20 mM, pH 6.0) or citrate buffer (20 mM, pH 6.0) containing 10 wt % DES (Bet: EG, 2:1), respectively. The far-UV CD spectrum of the treated samples was measured in a quartz cell with 0.1 mm optical path length in the range of 190–250 nm at 25 °C [25]. The near-UV CD spectrum was recorded at 25 °C from 250 to 320 nm with a 3.5 mL quartz cuvette [26]. The protein concentration was 0.02 g/L for measuring far-UV CD spectra, and the protein concentration was 0.05 g/L for measuring near-UV CD spectra. For each measurement, the according solvent was used as a blank for background subtraction.

## 3. Results and Discussion

### 3.1. Screening of Optimal DES for Enzymatic Reactions

**Figure 1:**
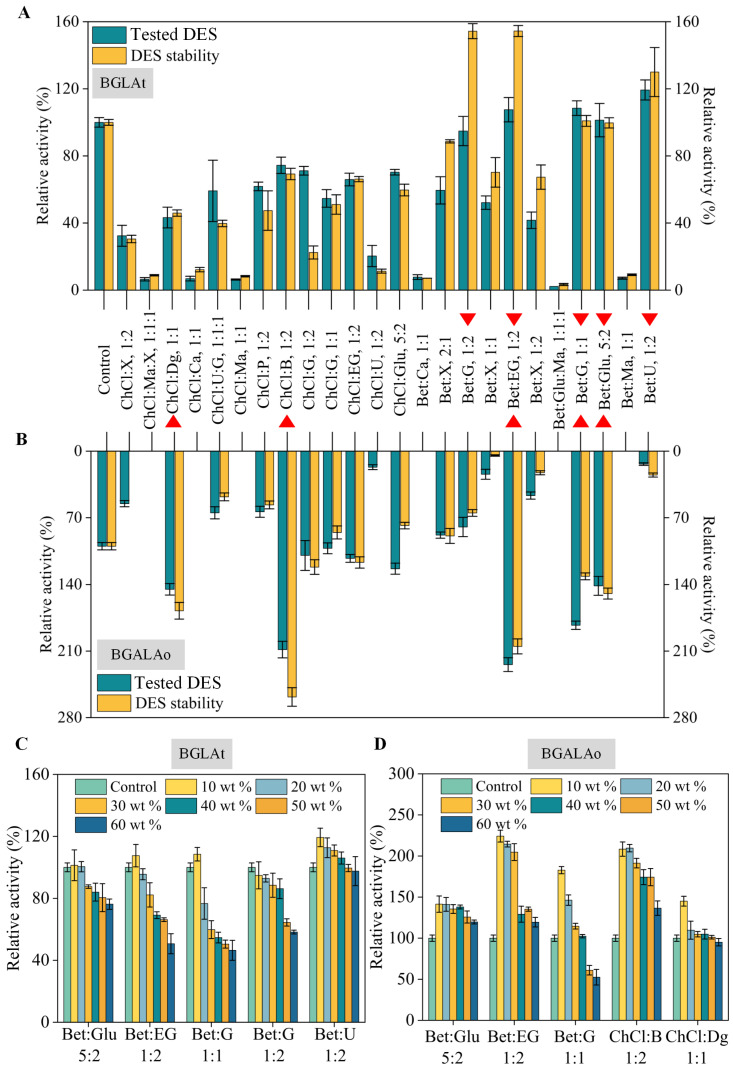
Effects of types and concentrations of DES on the activity and stability of combinatorial enzymes (BGLAt and BGALAo). (**A**) Study of BGLAt optimal DES (50 °C) and DES stability (50 °C, 1 h). (**B**) Study of BGALAo optimal DES (50 °C) and DES stability (50 °C, 1 h). The reaction medium is 10 wt % DES-buffer (citric acid buffer (20 mM, pH 6.0) contains 10 wt % of DES (Bet: EG, 1:2)) (**A**,**B**). (**C**) Effects of concentrations (10, 20, 30, 40, 50, and 60 wt %) of DES on the activity of BGLAt (50 °C). (**D**) Effects of concentrations (10, 20, 30, 40, 50, and 60 wt %) of DES on the activity of BGALAo (50 °C). The activity without DES, used as the control, was defined as 100% (**A**–**D**). Error bars represent the corresponding standard deviation of three independent experiments (*n* = 3).

Maintenance and enhancement of enzyme activity and catalytic efficiency by DES-based buffer solution was an effective method [14,27,28]. Therefore, 24 DES were prepared as an enzymatic reaction medium to facilitate the high-value transformation of GE (Figure S2). Previous studies have verified the capability of β-glucosidase from *A. tubingensis* [29] and β-galactosidase from *A. oryzae* [30] to hydrolyze ginsenosides. As shown in Figure 1A,B, the effect of DES on enzyme activity and stability was initially explored. BGLAt was more stimulated by betaine-based DES than choline chloride-based DES (Figure 1A). It could be that betaine does not react synergistically with polyols when combined to form DES, which preserve proteins from inactivation and aggregation [31]. In summary, 5 DES (Bet: G, 1:2; Bet: EG, 1:2; Bet: G, 1:1; Bet: Glu, 5:2; Bet: U, 1:2) were selected to evaluate the effect of DES concentration on BGLAt enzyme activity. There are few studies on the application of DES to β-glucosidase catalysis [15,32], and there is almost no report on the application of betaine-based DES. In the future, more examples of the effect of betaine-based DES on β-glucosidase activity should be discussed. In addition, there was no significant effect of the type of HBA on the activity for BGALAo (Figure 1B). However, the alcohol-based DES significantly activated β-galactosidase BGALAo, which is similar to the findings of previous studies [33,34]. The viscosity of U-based DES is higher than that of alcohol-based DES, and the high viscosity will impact the transfer rate between the enzyme and the substrate, thus reducing the catalytic efficiency of the enzyme [35]. Another possible reason is that polyols have a more powerful strength than U in forming H-bonds, which makes the substrate easily released from the hydrogen-bonding network and facilitates the combination of the substrate with the enzyme active center [36]. In summary, 5 DES (ChCl: Dg, 1:1; ChCl: B, 1:2; Bet: EG, 1:2; Bet: G, 1:1; Bet: Glu, 5:2) were selected to evaluate the effect of DES concentration on β-galactosidase activity.

Among the DES screened in the previous step, all three DES (Bet: G, 1:2; Bet: EG, 1:2; Bet: G, 1:1) promoted the activity of BGLAt and BGALAo. Bet: EG (1:2) was more effective for the activation of both enzymes (Figure 1C,D). A bell-shaped relationship was observed between the enzyme activity and the DES (Bet: EG, 1:2) concentration in the reaction system, with an optimum obtained when the volume of the added DES reached 10 wt %. Furthermore, the production of CK tended to decrease with the increase in DES (Bet: EG, 1:2) concentration, but the production of F_2_ was not affected (Figure S3). It is speculated that the excessive DES surrounds the active site of β-glucosidase and inhibits substrate entry, which is consistent with another study [37]. However, β-galactosidase was not affected by this. Overall, the production of De-g will be further improved by introducing solvent engineering in the combinatorial enzyme catalytic strategy. Therefore, 10 wt % Bet: EG (2:1) was chosen as a booster for the enzyme combinatorial catalysis in the next exploration.

### 3.2. Effects of DES (Bet: EG, 2:1) on Biocatalysts

**Figure 2:**
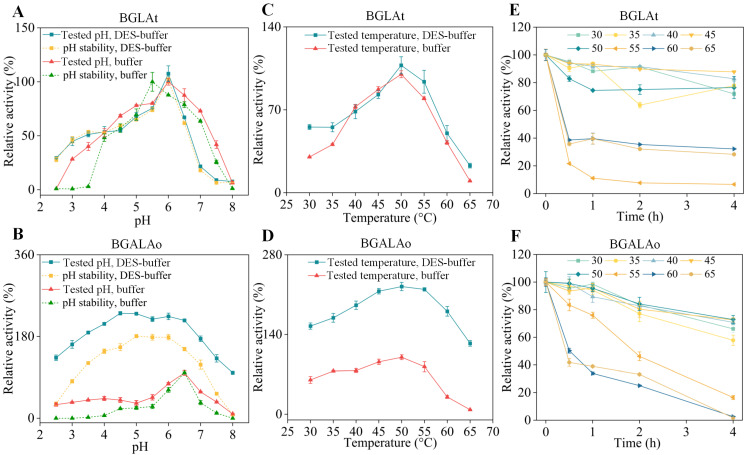
Effects of pH and temperature on the activity and stability of the combinatorial enzymes (BGLAt and BGALAo) incubated in the DES-buffer. (**A**) Study of the optimal pH (50 °C) and pH stability (50 °C, 1 h) of BGLAt incubated in the DES-buffer or buffer. (**B**) Study of the optimal pH (50 °C) and pH stability (50 °C, 1 h) of BGALAo incubated in the DES-buffer or buffer. (**C**) Study of the optimal temperature of BGLAt incubated in the DES-buffer or buffer. (**D**) Study of the optimal temperature of BGALAo incubated in the DES-buffer or buffer. The state (incubate in buffer) with the highest enzyme activity was defined as 100% (**A**–**D**). (**E**) Thermostability of BGLAt incubated in the DES-buffer or buffer. (**F**) Thermostability of BGALAo incubated in the DES-buffer or buffer. The activity without pre-incubation was defined as 100% (**E**,**F**). DES-buffer means that the buffer (citric acid buffer, 20 mM, pH 6.0) contains 10 wt % of DES (Bet: EG, 1:2) (**A**–**F**). Error bars represent the corresponding standard deviation of three independent experiments (*n* = 3).

It is well known that the optimum pH and temperature could make the enzymatic performance more effective [38,39]. Therefore, the effects of DES on the optimal pH and temperature of BGLAt and BGALAo were initially investigated. As shown in Figure 2A,B, the optimal pH of BGLAt was still 6.0, but the optimal pH of BGALAo was changed from 6.5 to 6.0 with the addition of DES compared to the buffer. In addition, the pH stability of BGLAt was improved when incubated in DES-buffer with a pH range of 2.5 to 4.0. Similarly, the activity and stability of BGALAo were significantly improved in the pH range from 2.5 to 8.0, even with 231.10% and 230.20% of the initial activity at pH 4.5 and 5.0. This is similar to many studies in which the solution containing DES significantly enhanced the activity and pH stability of the enzyme [40,41]. The improvement of the acid resistance of BGLAt and BGALAo makes it more widely used in industry. Moreover, the optimal temperature of BGLAt and BGALAo was not significantly changed with the DES addition compared to the buffer; for both, it was 50 °C (Figure 2C,D). After incubation at 50 °C for 4 h, BGLAt and BGALAo maintained over 70% of their initial activity (Figure 2E,F). However, there was a dramatic decrease in the activity of BGLAt and BGALAo once they exceeded 55 °C. Notably, the DES-added solution remarkably improved the thermostability of BGALAo, which contributed to the efficiency of the enzyme-based industrial process. Both BGLAt and BGALAo exhibit the highest activity at pH 6.0 and 50 °C, which provides the possibility for their co-catalytic reactions. To summarize, the reaction conditions of pH 6.0 and 50 °C were chosen for the next study of the DES-based high-value conversion of ginseng extracts.

### 3.3. Enzymatic Conversion of GE in the DES-Buffer

**Figure 3:**
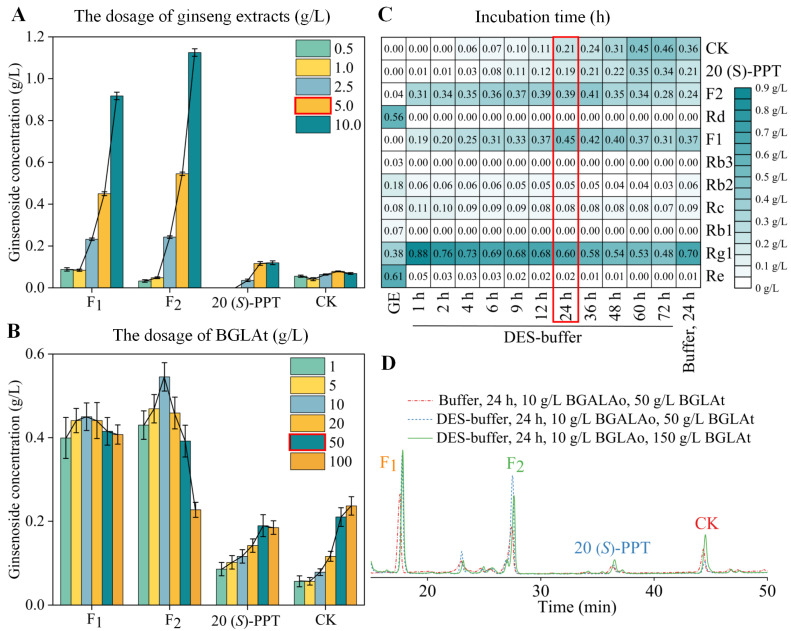
Process optimization for De-g production. (**A**) Substrate inhibition on De-g yield with different dosages of GE (0.5, 1.0, 2.5, 5.0, and 10.0 g/L) in DES-buffer. (**B**) Enzyme inhibition on De-g yield with different dosages of BGLAt (1, 5, 10, 20, 50, and 100 g/L) in DES-buffer. Error bars represent the corresponding standard deviation of three independent experiments (*n* = 3) (**A**,**B**). (**C**) De-g yield under the process (sampled at 1, 2, 4, 6, 9, 12, 24, 36, 48, 60, and 72 h), compared with GE and the samples in buffer for 24 h (buffer, 24 h). (**D**) HPLC analysis of De-g changes in samples containing 50 g/L BGLAt incubated in buffer, containing 50 g/L BGLAt incubated in 10 wt % DES-buffer, containing 150 g/L BGLAt incubated in 10 wt % DES-buffer. The remaining conditions for the preparation of samples were the same, containing 5 g/L GE and 10 g/L of BGALAo at 50 °C for 24 h reaction. De-g contains F_1_, F_2_, 20 (*S*)-PPT, and CK (**A**–**D**). DES-buffer means that the buffer (citric acid buffer, 20 mM, pH 6.0) contains 10 wt % of DES (Bet: EG, 1:2) (**A**–**D**).

The substrate concentration, the ratio of catalysts, and the duration of reaction are typical crucial factors influencing biocatalytic reactions. For a combined enzyme-catalyzed system with multiple substrates, these factors influence not only the reaction efficiency but also the product compositions [11]. Therefore, single factors (substrate concentration, enzyme concentration, and reaction time) were investigated for the optimization of the conversion conditions for converting ginseng extracts into De-g (F_1_, F_2_, 20 (*S*)-PPT, and CK) through a combined DES-based enzyme-catalyzed system. As shown in Figure 3A, the formation rate of De-g increased with a substrate concentration below 5 g/L. When the GE addition exceeded 5 g/L, the formation rates of F_1_ and F_2_ continued to increase, but the formation rates of 20 (*S*)-PPT and CK stabilized. In the combinatorial enzyme catalytic system, BGALAo converted the Ma-g (Rb_1_, Rc, Rb_2_, and Rb_3_) into F_1_ and F_2_, and BGLAt converted F_1_ and F_2_ into 20 (*S*)-PPT and CK (Figure S1C). It was indicated that BGALAo was not inhibited by GE concentrations over 5 g/L. Therefore, only the dosage of BGLAt was considered in the formation of De-g. As demonstrated in Figure 3B, F_1_ and F_2_ production had a bell-shaped variation, and the optimal level was achieved at a BGLAt dose of 10 g/L. The 20 (*S*)-PPT and CK were produced at the quickest rate when 50 g/L BGLAt was added. At that moment, De-g were in equilibrium with high F_1_, F_2_, 20 (*S*)-PPT, and CK levels. Thus, the optimal dosages of BGLAt and GE were 50 g/L and 5 g/L, respectively.

Based on the above analysis, the dynamic fluctuations of ginsenosides throughout the reaction process were studied. As the reaction progresses, the Ma-g (Re, Rg_1_, Rb_1_, Rc, Rb_2_, Rb_3_, and Rd) diminished, and De-g (F_1_, F_2_, 20 (*S*)-PPT, and CK) were generated. Notably, BGALAo could convert Re, Rb_1_, Rb_3_, and Rd into Rg_1_, F_1_, and F_2_ within 1 h (Figure S1C). After 24 h and 36 h of reaction, the production peaks for F_1_ and F_2_ were found, respectively. Furthermore, 20 (*S*)-PPT and CK production increased with reaction time, reaching 0.339 and 0.443 g/L at 72 h, respectively. To account for the time cost of industrial applications, it was recommended that the De-g were produced in 24 h. The sample after reacting in buffer for 24 h was used as a control (conditions were identical except for the reaction medium). Compared to buffer-reacted samples, DES-based combinatorial enzyme-catalyzed samples contained fewer Ma-g and more F_1_ and F_2_, which were 1.2–1.6 times. However, the production of 20 (*S*)-PPT and CK dropped to 0.21 and 0.29 g/L, respectively. It is speculated that DES did not change the conformation of BGALAo and may have made it easier for ginsenosides to bind to the enzyme active site. However, DES, although slightly activating BGLAt (Figure 2), may have covered up its active site or disrupted the conformation of its reaction. To make sure that the product had more F_1_, F_2_, 20 (*S*)-PPT, and CK, the dosage of BGLAt was increased (Figure 3D). Eventually, it would be the same as making 450 kg F1 ($ 140/g), 340 kg F2 ($ 140/g), 350 kg 20 (S)-PPT ($ 157/g), and 450 kg CK ($ 157/g) in 24 h from 5 tons of GE ($ 85/kg), which is very attractive for industrial production.

### 3.4. Extraction of Ginsenosides and Recycling of DES

**Figure 4:**
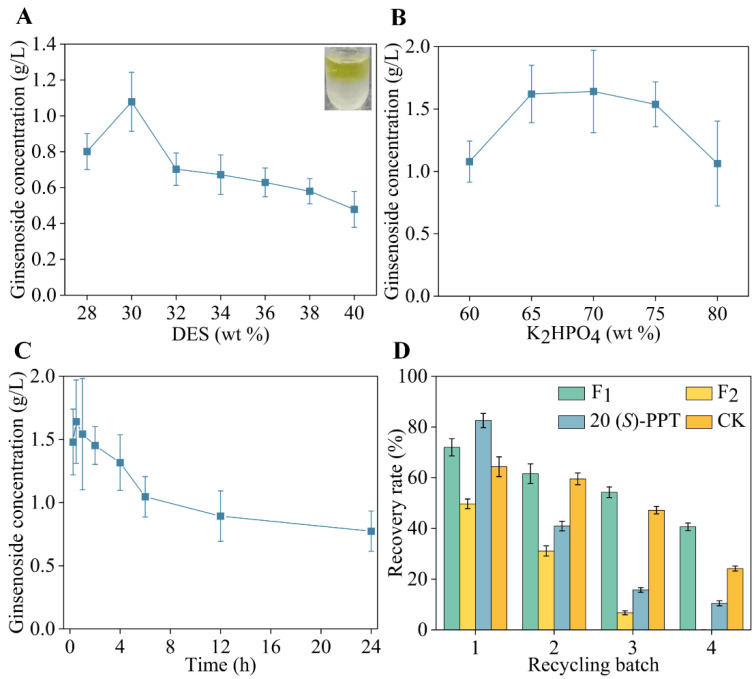
Optimization of ginsenosides extraction process by DES-based ATPS system. (**A**) Effects of the concentration (28, 30, 32, 34, 36, 38, and 40 wt %) of DES on the ATPS extraction process. (**B**) Effects of the concentration (60, 65, 70, 75, and 80 wt %) of K_2_HPO_4_ on the ATPS extraction process. (**C**) Effects of the extraction time (0.25, 0.5, 1, 2, 4, 6, 12, and 24 h) on the ATPS extraction process. (**D**) Recovery testing of De-g (F_1_, F_2_, 20 (*S*)-PPT, and CK) in multiple recycling batches.

The current work shows that the application of the novel solvent DES in the conversion of natural organic compounds is feasible, and it could further applied to ATPS for enriching products [16,42]. The DES-based phase diagram is necessary for the project of an aqueous two-phase extraction process of ginsenosides. As shown in Figure S4, most of the ginsenosides were concentrated in the upper phase of the ATPS system after phase-separation. The region above the binodal curve formed two phases, and the region below did not. A two-phase system based on Bet: EG/K_2_HPO_4_ could not be formed when the DES content was below 23%. DES concentration, salt concentration, and extraction time were further explored to enhance the efficiency of ginsenosides extraction by ATPS. The ginsenosides extraction efficiency gradually decreased when the concentration of DES was more than 30 wt % (Figure 4A). This may be because increasing the concentration of DES leads to the viscosity of top phase being increased, which impeded protein from transferring into the top phase. Thus, it suggested that 30 wt % was the optimal concentration of DES for ATPS, which was adopted in subsequent work. Furthermore, the analysis of the salt concentration showed that the optimal extraction efficiency was obtained when the K_2_HPO_4_ concentration was 70 wt %. Above that, the extraction efficiency went down as the viscosity of the bottom phase went up (Figure 4B). Therefore, the ideal salt content for the subsequent studies was established to be 70 wt %. Figure 4C depicts the temporal dependency of ginsenoside extraction efficiency. When the extraction time was 0.5 h, the upper phase had the highest concentration of ginsenosides. After that, the extraction efficiency decreased as extraction time increased. It was speculated that the substrates were taken up by the more viscous lower phase as the extraction time was prolonged. Therefore, 0.5 h was chosen as the appropriate extraction time. In conclusion, the developed DES-based ATPS has the benefits of being environmentally friendly, having a high extraction efficiency, and saving time.

The reuse of solvents is critical to the sustainable process. The recovered DES was utilized to catalyzed GE into De-g and build the ATPS system. As depicted in Figure 4D, the feasibility of a De-g recycling system was evaluated. It was observed that 71.98% of F_1_, 49.69% of F_2_, 82.55% of 20 (*S*)-PPT, and 64.33% of CK were recovered in the first batch. However, the recoveries of De-g were below 50% after the fourth batch biotransformation. Additionally, it was shown that DES-based ATPS could effectively recover the biocatalysts [43]. In the upper phase of the sample recovered by DES-ATPS after 24 h of reaction, 11% initial activity of BGALAo and 8% initial activity of BGLAt were found. If the CK-removed DES is re-recovered, and the appropriate amount of K_2_HPO_4_ and enzyme are added, another highly transforming ATPS circulation will be regenerated. DES-based ATPS is effective in recovering ginsenosides and biocatalysts, making the ATPS extractive conversion system more green and economical.

To date, multiple ginsenoside hydrolases have been developed and applied to the production of De-g [44,45]. However, exceeding the catalytic potential of enzymes, reusing catalytic media and biocatalysts are indicators that need to be perfected in scale-up production [46]. The addition of DES in the catalytic reaction solves these problems favorably, allowing for the incorporation of enzymatic catalysis and product extraction into one system. The DES-based ATPS developed by Han et al. recovered 75.79% of the catalytic product CK and 61.14% of the β-glucosidase from the top and bottom phases, respectively, and the recovered β-glucosidase could be recycled again for the preparation of CK [22]. In addition, the previous study showed that DES (prepared by betaine and polyols) has low toxicity towards the cell line (Caco-2 cells), bacteria, and plant seeds [47]. Therefore, the comprehensive resource utilization platform based on biocatalysis, reaction medium recovery, and product extraction in one is instructive for the green and economic production of De-g.

### 3.5. Characterization of the Effect of DES (Bet: EG 2:1) on Enzyme Structure

**Figure 5:**
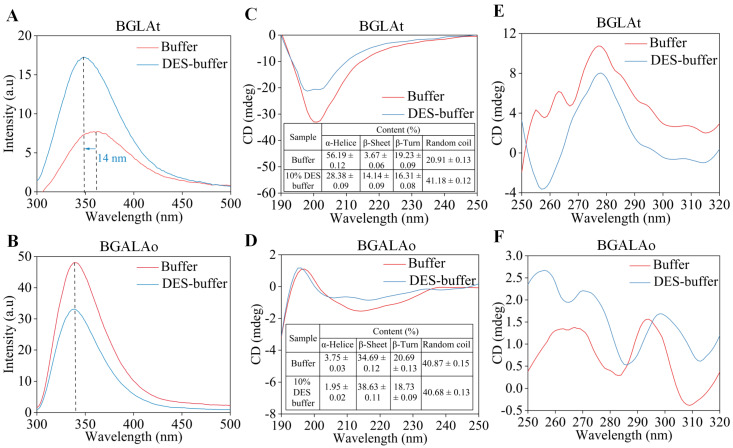
Spectra evaluating the impact of DES on the structure of the combinatorial enzymes (BGLAt and BGALAo). (**A**) Fluorescence spectra of BGLAt (0.5 µmol/L) in DES-buffer. (**B**) Fluorescence spectra of BGALAo (0.5 µmol·L^−1^) in DES-buffer. (**C**) Far-UV CD spectra of BGLAt (0.02 g/L) in DES-buffer. D Far-UV CD spectra of BGALAo (0.02 g/L) in DES-buffer. The tables show the effects of DES-buffer on the secondary structure content (%) of BGLAt (**C**) and BGALAo (**D**). (**E**) Near-UV CD spectra of BGLAt (0.05 g/L) in DES-buffer. (**F**) Near-UV CD spectra of BGALAo (0.05 g/L) in DES-buffer. The samples of BGLAt and BGALAo are incubated in buffer as control, respectively (**A**–**F**). DES-buffer means that the buffer (citric acid buffer, 20 mM, pH 6.0) contains 10 wt % of DES (Bet: EG, 1:2) (**A**–**F**).

In order to gain insight into the effect of DES on proteins, fluorescence spectroscopy and CD spectroscopy analysis were performed. Fluorescence of protein originates from its intrinsic fluorophore amino acids, e.g., phenylalanine (Phe), tyrosine (Tyr), and tryptophan (Trp) [48]. Among them, the maximal emission wavelength (*λ*_max_) of Trp in water is near 350 nm and is highly volatile with the change of local environment. As shown in Figure 5A,B, the *λ*_max_ of BGLAt and BGALAo in buffer were around 350 nm, which is similar to a previous study [41,49]. However, the sample of BGLAt exhibited a blue shift of 14 nm after adding DES, which suggested a reduction in microenvironmental hydrophobicity of the aromatic amino acid residues. In addition, an increase in the intrinsic fluorescence intensity of BGLAt was observed due to the compactness of the structure [26]. These findings suggest that the stabilizing effects of polyol-class solvents on BGLAt might be related to the conformational changes around its active site rather than to DES interactions with the active site. In contrast, the *λ*_max_ of BGALAo did not change after adding DES, but its fluorescence maximal intensity (*I*_max_) showed a decreasing trend. It was possible that the gradual formation of the polymer of DES with BGALAo led to changes in the microenvironment of tryptophan residues and occlusion, which reduced the fluorescence intensity.

In addition, CD spectroscopy is a powerful technique for analyzing the secondary and tertiary structures of proteins [50], which are examined in the far-UV (≤250 nm) and near-UV (>250 nm) regions, respectively. As demonstrated in Figure 5C and D, BGLAt and BGALAo showed a decrease in the α-helix content and an increase in the β-sheet content of the protein’s secondary structure after adding DES. Several research groups have suggested that the reduction in α-helix content and increase in β-sheet content might affect the enzyme active region and improve its activity [32]. This verified the previous findings that the addition of DES increased the activity of BGLAt and BGALAo (Figure 1). Changes in the tertiary structure of BGLAt and BGALAo were observed in the near-UV region between 250 and 320 nm, and the spectrograms are shown in Figure 5E and F. The samples of BGLAt all exhibited positive peaks at 275 nm, which is characteristic of aromatic amino acid residues. In addition, the peak shape of BGLAt at 270–320 nm did not change after adding DES, but a signal weakening was observed. It has been shown that a decrease in the signal is known to be characteristic of a loss in the protein stability [51]. In addition, the BGLAt samples exhibited considerably different peak shapes between 250 and 270 nm, suggesting a shift in the protein’s tertiary structure, which is consistent with the fluorescence spectroscopy data. The addition of DES changed the microenvironment of the tryptophan residues of BGLAt. Moreover, ethylene glycol with two hydroxyl groups would bind to protein via more hydrogen bonds, so the structure becomes more rigid and stable. Whereas ginsenosides have a sprawling structure, the rigid active center limits the enzyme interactions with the substrate. This also explains that although BGLAt was activated, its hydrolysis efficiency of ginsenosides became lower. However, for BGALAo, although the peak shape changed slightly, the addition of DES enhanced the intensity of the signal. It was shown that the increase in signal might be associated with the increase in enzyme stability [51]. In addition, the minimal value of BGALAo was at 280 nm, and the maximum value was at 255 nm after adding DES, which could be the characteristic peak of a disulfide bond [41]. The addition of DES may contribute to the formation of disulfide bonds and Tyr residues by BGALAo. The more disulfide bonds, the stronger the protein molecule is against environmental changes. The addition of DES not only remarkably stimulated the activity of BGALAo, but also greatly improved its hydrolysis efficiency of ginsenosides, which is instructive for the production of De-g.

## 4. Conclusions

In this work, we investigated the combinatorial enzymatic conversion of ginsenosides in DES buffer. Bet: EG, which was synthesized from betaine and ethylene glycol, significantly activated the activity of β-glucosidase and β-galactosidase, and it also improved the acid resistance and temperature stability of the enzymes. Five grams of ginseng extracts in DES-buffer could be converted to 1.24 g of De-g (F_1_, F_2_, 20 (*S*)-PPT, CK), which was 1.1 times that of the buffer sample. On the other hand, the constructed DES-based ATPS could rapidly recover DES and more than 60% of De-g from the top phase. These results indicate that DES is an efficient and sustainable conversion medium that can efficiently convert value-added ginseng extracts to De-g and can be applied to green extraction of natural products.

## Figures and Tables

**Table 1:** The molar ratio of the hydrogen bond acceptors (HBA) and hydrogen bond donors (HBD) for DES preparation.

Hydrogen Bond Acceptor (HBA)	Hydrogen Bond Donor (HBD)		Molar Ratio	Abbreviation
	HBD1	HBD2		
Choline chloride	1,2-Propanediol		1:2	ChCl:P
	Urea		1:2	ChCl:U
	Ethylene glycol		1:2	ChCl:EG
	1,4-Butanediol		1:2	ChCl:B
	Glycerol		1:1	ChCl:G
	Glycerol		1:2	ChCl:G
	Citric acid		1:1	ChCl:Ca
	D-Glucitol		1:1	ChCl:Dg
	Malic acid		1:1	ChCl:Ma
	Xylitol		1:2	ChCl:X
	Glucose		5:2	ChCl:Glu
	Malic acid	Xylitol	1:1:1	ChCl:Ma:X
	Urea	Glycerol	1:1:1	ChCl:U:G
Betaine	Xylitol		1:2	Bet:X
	Xylitol		1:1	Bet:X
	Xylitol		2:1	Bet:X
	Glycerol		1:1	Bet:G
	Glycerol		1:2	Bet:G
	Citric acid		1:1	Bet:Ca
	Ethylene glycol		1:2	Bet:EG
	Urea		1:2	Bet:U
	Malic acid		1:1	Bet:Ma
	Glucose		5:2	Bet:Glu
	Malic acid	Glucose	1:1:1	Bet:Ma:Glu

## Data Availability

The date are available from the corresponding author.

## Supplementary Materials

The following supporting information can be downloaded at: https://www.mdpi.com/article/10.3390/foods12030496/s1, Figure S1: (A) SDS PAGE analysis of the BGLAt and BGALAo. M, 25 1 8 0 kDa protein marker; C, acetate buffer 20 mM, pH 6.0 1, BGLAt; 2, BGALAo. (B) HPLC analysis of samples of lyophilized enzymes (30 0 U/mL BGLAt, 17 0 U/mL BGALAo, and the c ombinatorial enzymes contained 300 U/mL BGLAt and 17 0 U/mL BGALAo) reacted with the final concentration of 5 g/L GE in 20 mM acetate buffer (pH 6.0) at 50 °C, 200 rpm for 24 h, The samples were extracted with an equal volume of n butanol, and the top phase (water saturated n butanol fraction) was were evaporated and re dissolved with 100% methanol. The re dissolved solution was filtered and detected by HPLC. (C) The biotransformation pathways of ginsenosides involved in the reactions; Figure S2. 24 combinations of DES. Abbreviations reference Table 1; Figure S3. HPLC analysis of the effect of DES concentration on the conversion of ginsenosides DES was prepared by betaine and ethylene glycol at a molar ratio of 1:2. 300 U/mL BGLAt and 170 U/mL BGALAo reacted with the final concentration of 5 g/L GE in d ifferent concentrations 10, 20, 30, 40, 50, and 60 wt %) of DES buffer 20 mM pH 6.0 citrate buffer) at 50 °C, 200 rpm for 24 h, The samples were extracted with an equal volume of n butanol, and the top phase (water saturated n-butanol fraction) was were evaporated and re dissolved with 100% methanol. There dissolved solution was filtered and detected by HPLC; Figure S4. Phase diagram of the DES based ATPS. DES was prepared by betaine and ethylene glycol at a molar ratio of 1:2.

## Abbreviations

Ma-g: major ginsenosides; De-g, deglycosylated ginsenosides; DES, deep eutectic solvents; ATPS, aqueous two-phase system; GE, ginseng extracts.
