# Supplementary material for: High-Value Bioconversion of Ginseng Extracts in Betaine-Based Deep Eutectic Solvents for the Preparation of Deglycosylated Ginsenosides
Source: Foods. 2023 Jan 20;12(3):496. doi: 10.3390/foods12030496 (PMC9914094; doi:10.3390/foods12030496)
Supplement: Supplementary file 1 [file foods-12-00496-s001.zip › Supplementary material.pdf]

Supplementary material

High-value bioconversion of ginseng extracts in betaine-based deep eutectic solvents for the preparation of deglycosylated ginsenosides

Wenhua Yang[1], Qiuya Gu[1], Jianli Zhou[2], Xiaobo Liu[3], Xiaobin Yu[1, *]

[1]Key Laboratory of Industrial Biotechnology, Ministry of Education, School of Biotechnology, Jiangnan University, Wuxi 214000, Jiangsu, China

[2]Guizhou Province Key Laboratory of Fermentation Engineering and Biopharmacy, School of Liquor and Food Engineering, Guizhou University, Guiyang 550003, Guizhou, China

[3]School of Environmental and Biological Engineering, Nanjing University of Science and Technology, Nanjing 210094, Jiangsu, China

*Corresponding author

Email: xbyu@jiangnan.edu.cn

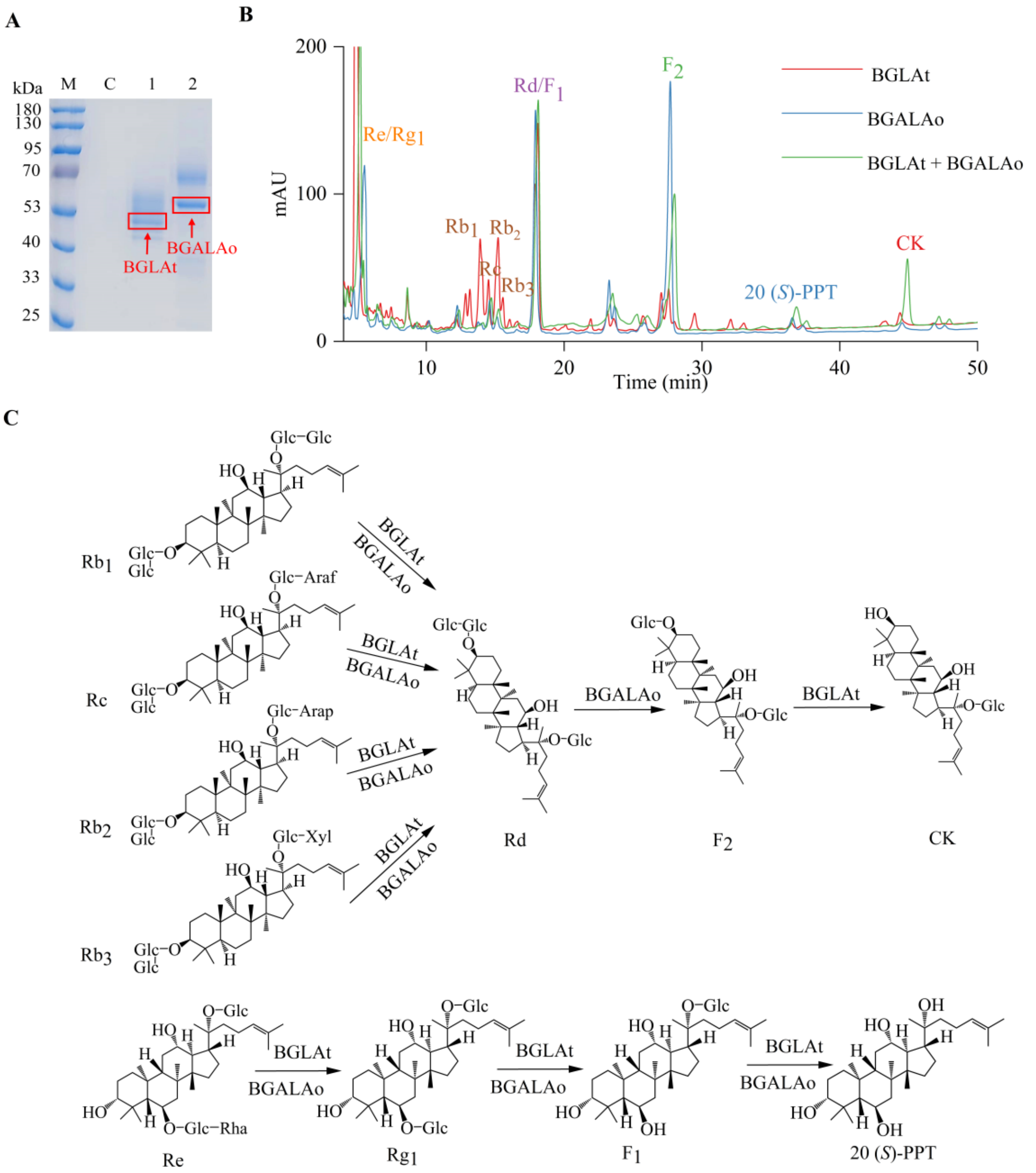

Figure S1. (A) SDS-PAGE analysis of the BGLAt and BGALAo. M, 25-180 kDa protein marker; C, acetate buffer (20 mM, pH 6.0); 1, BGLAt; 2, BGALAo. (B) HPLC analysis of samples of lyophilized enzymes (300 U/mL BGLAt, 170 U/mL BGALAo, and the combinatorial enzymes contained 300 U/mL BGLAt and 170 U/mL BGALAo) reacted with the final concentration of 5 g/L GE in 20 mM acetate buffer (pH 6.0) at 50 ℃, 200 rpm for 24 h, respectively. The samples were extracted with an equal volume of n-butanol, and the top phase (water-saturated n-butanol fraction) was were evaporated and re-dissolved with 100% methanol. The re-dissolved solution was filtered and detected by HPLC. (C) The biotransformation pathways of ginsenosides involved in the reactions.

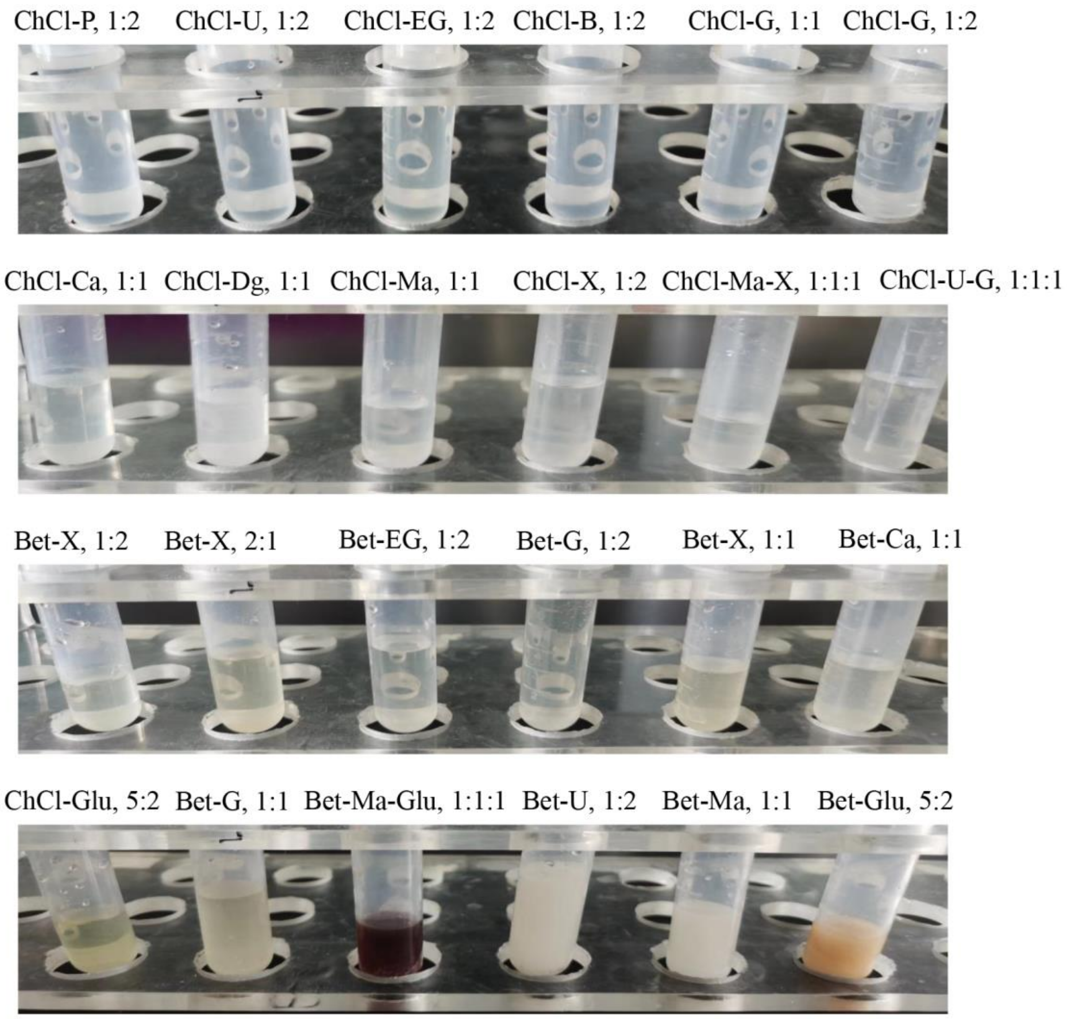

Figure S2. 24 combinations of DES. Abbreviations reference Table 1.

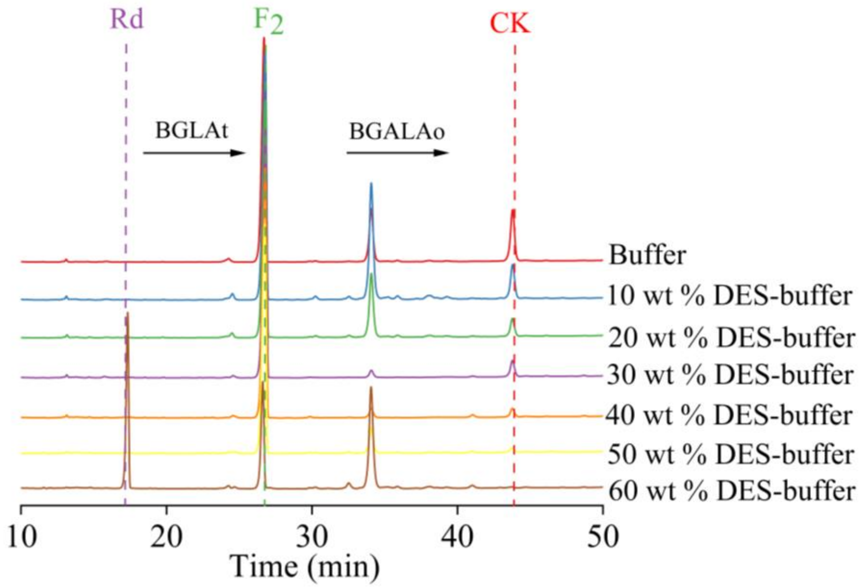

Figure S3. HPLC analysis of the effect of DES concentration on the conversion of ginsenosides. DES was prepared by betaine and ethylene glycol at a molar ratio of 1:2. 300 U/mL BGLAt and 170 U/mL BGALAo reacted with the final concentration of 5 g/L GE in different concentrations (10, 20, 30, 40, 50, and 60 wt %) of DES-buffer (20 mM, pH 6.0 citrate buffer) at 50 ℃, 200 rpm for 24 h, respectively. The samples were extracted with an equal volume of n-butanol, and the top phase (water-saturated n-butanol fraction) was were evaporated and re-dissolved with 100% methanol. The re-dissolved solution was filtered and detected by HPLC.

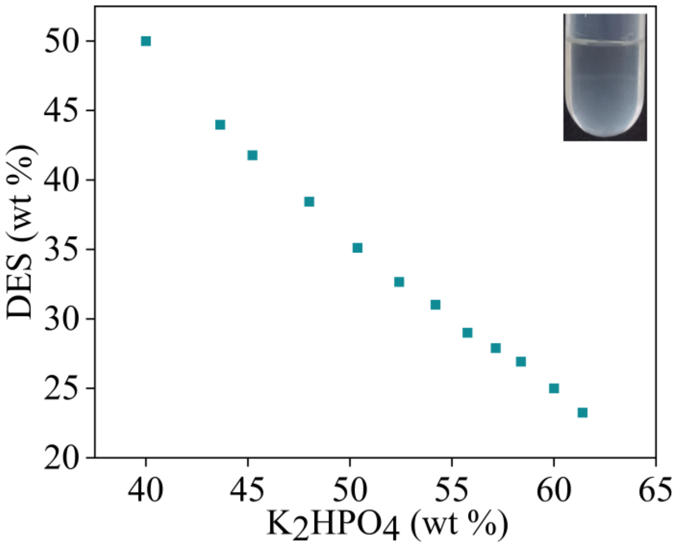

Figure S4. Phase diagram of the DES-based ATPS. DES was prepared by betaine and ethylene glycol at a molar ratio of 1:2.
